# Cellular Proteins in Influenza Virus Particles

**DOI:** 10.1371/journal.ppat.1000085

**Published:** 2008-06-06

**Authors:** Megan L. Shaw, Kathryn L. Stone, Christopher M. Colangelo, Erol E. Gulcicek, Peter Palese

**Affiliations:** 1 Department of Microbiology, Mount Sinai School of Medicine, New York, New York, United States of America; 2 Northeast Biodefense Center Proteomics Core, W.M. Keck Foundation Biotechnology Laboratory, Yale University, New Haven, Connecticut, United States of America; 3 Department of Medicine, Mount Sinai School of Medicine, New York, New York, United States of America; Oregon Health & Science University, United States of America

## Abstract

Virions are thought to contain all the essential proteins that govern virus egress from the host cell and initiation of replication in the target cell. It has been known for some time that influenza virions contain nine viral proteins; however, analyses of other enveloped viruses have revealed that proteins from the host cell can also be detected in virions. To address whether the same is true for influenza virus, we used two complementary mass spectrometry approaches to perform a comprehensive proteomic analysis of purified influenza virus particles. In addition to the aforementioned nine virus-encoded proteins, we detected the presence of 36 host-encoded proteins. These include both cytoplasmic and membrane-bound proteins that can be grouped into several functional categories, such as cytoskeletal proteins, annexins, glycolytic enzymes, and tetraspanins. Interestingly, a significant number of these have also been reported to be present in virions of other virus families. Protease treatment of virions combined with immunoblot analysis was used to verify the presence of the cellular protein and also to determine whether it is located in the core of the influenza virus particle. Immunogold labeling confirmed the presence of membrane-bound host proteins on the influenza virus envelope. The identification of cellular constituents of influenza virions has important implications for understanding the interactions of influenza virus with its host and brings us a step closer to defining the cellular requirements for influenza virus replication. While not all of the host proteins are necessarily incorporated specifically, those that are and are found to have an essential role represent novel targets for antiviral drugs and for attenuation of viruses for vaccine purposes.

## Author Summary

Viruses are released from infected cells in the form of virions, which contain all the essential factors necessary for initiating infection in a new target cell. For influenza virus, it is known that virions contain the viral genome, a lipid envelope, and at least nine viral proteins. We performed a detailed proteomic analysis of purified influenza virus particles using mass spectrometry and database searching for protein identification, and in addition to the nine viral proteins, we identified 36 host proteins. These host proteins are present both inside the influenza virus particle and on the viral envelope. All viruses require host cell factors to complete their replication cycles, and they also have to contend with the antiviral defense mechanisms of the host. Virus–host interactions may therefore provide the key to understanding viral pathogenesis and may also present us with new targets for the design of antiviral drugs. For influenza virus, information on the requirement of cellular factors is limited, but the description of these 36 host proteins that are packaged into the virion provides a foundation for further analysis into the involvement of these cellular pathways in the influenza virus life cycle.

## Introduction

Knowledge of the protein composition of a virus particle often serves as an initial guide in determining functional roles for viral proteins. Virion proteins are commonly termed “structural proteins” and broadly-speaking, include proteins that either form an integral part of the virus architecture or are required for the first round of genome replication. This view of a virion being a minimal package of genome and essential viral proteins is now being challenged due to enhanced proteomics techniques and the availability of annotated genomic sequences for several mammalian species. These advances have extended proteomic analyses of virions to include host proteins that may be packaged into the virus particle along with the viral components. Enveloped viruses in particular have the capability of incorporating numerous host proteins, both into the interior of the virus particle as well as into the lipid envelope [1],[2]. Several proteomic studies on herpesviruses have been undertaken, the majority of which focused on correctly identifying the viral constituents of the virion but many also reported finding cellular proteins [3]–[9]. Similarly, host proteins have been detected in vaccinia virions [10]. For RNA viruses, extensive proteomic analysis has been performed on human immunodeficiency virus type 1 (HIV-1) and Moloney murine leukemia virus (MoMLV) vector particles, and they too have been found to incorporate numerous cellular proteins [11]–[13].

For the most part the functional significance of these packaged host proteins has not yet been determined but some proteins are known to interact specifically with a viral protein and this has enabled the significance of their incorporation to be studied in more depth. These include Tsg101, cyclophilin A and APOBEC3G, all of which are packaged into HIV-1 virions [11], [12], [14]–[17]. Tsg101 plays a crucial role in virus assembly [14],[18], cyclophilin A modulates HIV-1 infectivity [19] and APOBEC3G is an anti-viral factor that promotes hypermutation of the viral genome [20]. These three proteins alone have significantly added to the understanding of how HIV-1 interacts with its host and they serve as an example of what can be learned from studying virion-associated host proteins. Although there are descriptions of interactions between certain cellular proteins and individual influenza virus proteins, for the most part this has not been done in a comprehensive manner and comparatively little is known about the requirement for host cell factors during the different stages of the influenza virus life cycle. In an effort to discover host factors involved particularly in genome replication, proteomic analyses of native influenza virus ribonucleoprotein and polymerase complexes have been performed which resulted in the identification of 45 interacting cellular proteins [21]. It is anticipated that cellular proteins found within the influenza virus particle may provide clues as to the virus assembly pathway and also early events that govern virus infectivity.

Of the eleven influenza A virus encoded proteins, nine have been identified in the virion [22]. The exceptions being NS1 and PB1-F2, the latter of which is not encoded by all influenza A viruses. The glycoproteins hemagglutinin (HA) and neuraminidase (NA) are embedded into the lipid envelope of the virus particle and form the characteristic spikes visible under the electron microscope [23]–[25]. Another membrane protein, the ion channel protein M2 is also found within the virion but at significantly lower levels than HA or NA [26]. The matrix protein M1 lies beneath the viral membrane and surrounds the eight ribonucleoprotein (RNP) segments, which consist of viral RNA coated with the nucleoprotein (NP) and bound by the trimeric polymerase complex (PB1, PB2, PA) [25],[27]. Finally the nuclear export protein (NEP) is also found within influenza virions [28]. The majority of these proteins were identified on the basis of size by polyacrylamide gel electrophoresis but because detection of proteins by this method is restricted to more highly abundant proteins, the presence of M2 and NEP proteins in the influenza virion was only discovered much later using specific antibodies [26],[28]. Any cellular proteins that may be incorporated into viral particles are also likely to be present at very low levels and while antibody-mediated detection is extremely sensitive, it is not practical when analyzing complexes of unknown composition. Mass spectrometry of tryptic peptides combined with database searching for identification is now the preferred method for such proteomic studies. In this report we utilize two complementary mass spectrometry techniques to analyze the protein content of purified influenza virus particles and specifically, to identify incorporated cellular proteins. Our analysis resulted in the identification of 9 virus-encoded proteins and 36 host-encoded proteins.

## Results

### Purification of influenza virus

**Figure 1:**
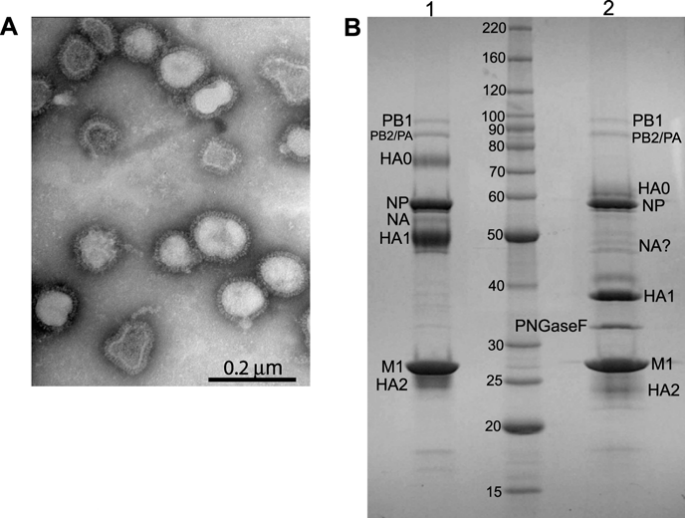
Analysis of purified influenza virus preparations. (A) Electron micrograph of negatively stained, sucrose gradient purified influenza A/WSN/33 virus at 50,000× magnification. (B) SDS-PAGE separation of proteins in a purified influenza virus preparation. 15 ug of untreated (lane 1) or deglycosylated (lane 2) proteins were separated on an 8–16% polyacrylamide gel and stained with Coomassie blue. The positions of the viral proteins, identified by their predicted molecular weights, are indicated.

Virion proteomic analysis requires a highly purified preparation of virus and the choice of host cell used for virus growth is also an important consideration. While MDCK (Madin Darby canine kidney) cells are the preferred cell line for growth of influenza virus in tissue culture, the dog genome is not yet fully annotated and this would restrict the identification of cellular proteins. For the same reason, virus grown in embryonated chicken eggs was also not the best option. As a compromise between cells that would support high levels of virus growth and cells that could be used to search the most extensive protein database (i.e. human), Vero (African green monkey kidney) cells were selected as the host cell line. There are a growing number of non-human primate sequences in the NCBI database and because of significant homology between primate and human proteins the human protein database could be used to identify incorporated host proteins. For later comparison, smaller amounts of virus were also purified from infected A549 (human carcinoma lung epithelial) cells. Supernatant collected from Vero cells infected with influenza A/WSN/33 virus was first clarified and the virus was concentrated through a sucrose cushion before being purified over a 30–60% sucrose gradient. The purity of the virus preparation was assessed by electron microscopy following negative staining (Fig. 1A). Both intact influenza virions and partially disrupted virions were observed but importantly, there was no obvious contamination with cellular material. The proteins in the purified virus preparation were separated by SDS-PAGE and stained with Coomassie blue, and for identification of the viral glycoproteins, a deglycosylated sample was compared to an untreated sample (Fig. 1B). All major viral proteins were visible. The three polymerase proteins resolved as two bands, both uncleaved (HA0) and cleaved (HA1 and HA2) forms of HA were present, as were bands consistent with the molecular weights for NP, NA and M1. There were also some much fainter bands visible that may represent cellular proteins.

### Proteomic analysis of influenza virions

The ability to fractionate protein samples to enhance the dynamic range of detectable proteins is a key issue when identifying the components of a protein complex by mass spectrometry. For this study two complementary techniques were used, one of which is based on separation of proteins and the other on separation of peptides. For the first method, both glycosylated and deglycosylated virus preparations were separated by SDS-PAGE on an 8–16% gradient gel (Fig. 1B). Deglycosylation is required for several reasons: Firstly, because trypsin does not always efficiently digest highly glycosylated proteins and, secondly, because unmodified peptides generally have higher electrospray ionization efficiencies than their glycosylated counterparts. Finally, because deglycosylation produces a more uniform set of peptides from a potentially diverse number of glycoprotein isoforms, the sensitivity is increased. That said, in this study we did not find that deglycosylation increased the number of proteins identified (see Tables S1 and S2 for a comparison) and therefore the reported identifications from the two approaches were combined. Following Coomassie blue staining, each lane was cut into successive slices from top to bottom and the individual slices were subjected to in-gel trypsin digestion. This procedure was repeated on a 20% gel and gel slices less than 25 kDa were excised, so as to maximize the chances of detecting small molecular weight proteins. The peptides in each gel slice were then analyzed by liquid chromatography tandem mass spectrometry (LC-MS/MS) and the resulting fragment ion spectra were searched against protein databases for identification.

The second method employed in this study was multidimensional protein identification technology (MudPIT). A deglycosylated purified virus preparation was digested with trypsin *en masse* and the peptides in the mixture were separated by two dimensional chromatography, first on the basis of charge and then on hydrophobicity. The second chromatography separation step was directly coupled to the mass spectrometer detector and the resulting spectra were searched against the database for protein identification. The disadvantage of MudPIT is that there is no information on the size of the proteins which is useful for confirmation of protein identity. However, the method allows for the detection of low abundance proteins and extremely small molecular weight proteins that are often lost during gel-separation or gel-extraction steps.

### Identification of influenza virus-encoded virion proteins

All nine virus-encoded proteins previously described to be in the influenza virion were identified by both MS methods (Table 1). These are PB1, PB2, PA, HA, NP, NA, M1, M2 and NEP. Peptides from NS1 or PB1-F2 were not detected. Table 1 lists the predicted mass of each protein, the gel slice in which it was detected, the number of observed peptides and the percent sequence coverage of the protein. The statistical score associated with the match is also noted. MASCOT scores are used for the SDS-PAGE and LC-MS/MS analysis, while protein prophet scores are used for the MudPIT analysis. The HA, NP, NA and M1 proteins were all found in multiple gel slices. For HA, this was expected due to the presence of uncleaved HA0 as well as the cleaved sub-units HA1 and HA2. However for both HA and particularly NP and M1, the proteins appear to be distributed over a wider-than-expected size range. This perhaps reflects the fact that they are predicted to be the most abundant proteins in the influenza virion [27] and these amounts may exceed the resolving capacity of the gel, causing them to smear. From their predicted size, PB1 and PB2 are expected to migrate together, however in fact we found that PB1 migrates slower than PB2, which resolves together with PA. This is in agreement with the first mapping data for the assignment of protein products to RNA segments [29] but the reason for the different migration patterns of PB1 and PB2 is still not known. Generally, the sequence coverage for each protein, which represents the number of unique peptides identified, was greater with the gel-fractionation and LC-MS/MS analysis. The exceptions are HA and NA, where greater sequence coverage was obtained with the MudPIT analysis.

**Table 1:** Virion-associated influenza virus proteins identified by mass spectrometry.

		SDS-PAGE and LC-MS/MS Analysis				MudPIT Analysis		
Protein name	Mass (Da)	Gel slice^a^	No. of observed peptides^c^	Mascot score^e^	Sequence coverage (%)^f^	No. of observed peptides^c^	Protein prophet score^e^	Sequence coverage (%)^f^
**PB1**	86516	10	35	700	37.1	5	1	8.6
**PB2**	85796	11	34	768	35.6	17	1	23.7
**PA**	82531	11	23	458	28.2	5	1	9.4
**HA**	63525	11–17,21–24,26–34,38–43,48	2–70^d^	54–546^d^	29.4	22	1	40.4
**NP**	56244	15–19,23,27,30	5–61^d^	66–1073^d^	46.8	32	1	35.3
**NA**	49689	20–21,28	3–6^d^	52–98^d^	15.5	9	1	22.7
**M1**	27864	32–46	3–102^d^	76–787^d^	66.3	12	0.98	34.7
**M2**	11313	46–47	2–3^d^	39–101^d^	48.5	1	0.96	11.3
**NEP**	14327	15^b^	7	77	31.4	1	0.95	7.4

a Gel slices were numbered consecutively from the top to the bottom of an 8–16% gel.

b From a higher percentage gel.

c Observed peptides include all peptides that differ either by sequence, modification or charge.

d Values represent the range when the protein was found in multiple gel slices.

e A Mascot score ≥50 and a Protein prophet score ≥0.95 are equivalent (p<0.05).

f Sequence coverage is based on peptides with unique sequence.

### Identification of virion-associated cellular proteins

In total, we identified 36 cellular proteins in the purified influenza virus preparation. Seventeen of these were identified by both MS methods (Table 2), another 13 were identified only with the MudPIT analysis (Table 3) and 6 were identified only with the gel-fractionation and LC-MS/MS analysis (Table 4). Each table indicates the protein name, its predicted mass, the gel slice in which it was found (where relevant), the number of observed peptides, the score associated with the match and the percent sequence coverage. In addition, the predicted cellular localization of the protein is shown along with its abundance at the transcript level. Abundance in the kidney is noted because of the use of Vero cells, while abundance in the lung is more biologically relevant for influenza virus. The final column lists other viruses that have been reported to incorporate the observed cellular protein into their virions.

**Table 2:** Cellular proteins identified in purified influenza virions by both gel fractionation and MudPIT LC-MS/MS analyses.

Protein Name	Entrez Gene ID	Mass (Da)	SDS-PAGE and LC-MS/MS Analysis				MudPIT Analysis			Cellular localization	Expression profile (TPM)^f^		Reported in other viruses
			Gel slice^a^	No. of observed peptides^b^	MASCOT score^d^	sequence coverage (%)^e^	No. of observed peptides^b^	Protein prophet score^d^	sequence coverage (%)^e^				
											Kidney	Lung	
**pyruvate kinase**	5315	57878	17	6	78	11.9	4	1	10.9	cytoplasm	2961	1701	KSHV [8], HIV-1 [11]
**beta tubulin**	203068	47767	19*	27	555	35.9	2	1	9.8	cytoplasm	1246	1057	HCMV [3], EBV [6], VV [10], MoMLV [13]
**alpha tubulin**	7846	50158	18–19*	3–7^c^	186–314^c^	20.6	2	0.99	8.1	cytoplasm	1795	3792	HCMV [3], VV [10], HIV-1 [11]
**enolase 1**	2023	47169	20*	6	318	22.1	4	1	9	cytoplasm	3325	2135	HCMV [3], EBV [6], KSHV [8]
**beta actin**	60	41005	22–23*	5–14^c^	158–372^c^	37.5	2	0.92	8	cytoplasm	3117	3708	HCMV [3], EBV [6], VV [10], HIV-1 [11],[31], KSHV [8], MoMLV [13]
**annexin A1**	301	38714	28	6	90	21.7	3	1	14.7	cytoplasm/ membrane	90	327	HCMV [3], VV [10], HIV-1 [11]
**glyceraldehyde-3-phosphate dehydrogenase**	2597	36054	28	5	69	17.9	1	1	16.3	cytoplasm	2340	4167	HCMV [3], HIV-1 [11],[12], KSHV [8], hPIV3 [85], MoMLV [13]
**annexin A2**	302	38576	29	17	600	46.3	13	1	40.4	cytoplasm/ membrane	578	828	HCMV [3], VV [10], KSHV [8], HIV-1 [11]
**tropomyosin 1**	7168	32876	29–30	3–10^c^	75–233^c^	34.2	2	0.99	7	cytoplasm	331	163	
**glypican 4 (k-glypican)**	2239	62398	30	3	63	5	1	0.97	3.1	membrane	80	17	HIV-1 [11]
**tropomyosin 3**	7170	27175	31	14	357	37.5	2	1	7	cytoplasm	251	324	
**annexin A4**	307	35883	31	10	181	39.5	2	1	6.7	cytoplasm/ membrane	227	122	
**CD9**	928	25431	39*	5	74	7.5	2	1	15.4	membrane	113	190	HIV-1 [11], MoMLV [13]
**CD81**	975	25813	2#	2	48§	8.5	3	1	16.9	membrane	85	312	HIV-1 [11], MoMLV [13], VV [82]
**cofilin 1**	1072	18502	44	11	318	50.6	3	1	35.6	cytoplasm	431	899	HCMV [3], EBV [6], HIV-1 [11],[31]
**cyclophilin A**	5478	18012	46	5	109	18.8	2	1	12.9	cytoplasm	530	1030	HIV-1 [11],[12],[15],[16], HCMV [3], VV [10], VSV [76], KSHV [8]
**profilin**	5216	15054	13#	3	44§	20	1	0.85	11.6	cytoplasm	203	259	HIV-1 [11]

a Gel slices were numbered consecutively from the top to the bottom of an 8–16% gel.

# From a higher percentage gel.

*: Best sequence coverage was obtained with the deglycosylated sample.

b Observed peptides include all peptides that differ either by sequence, modification or charge.

c Values represent the range when the protein was found in multiple gel slices.

d A Mascot score ≥50 and a Protein prophet score ≥0.95 are equivalent (p<0.05).

**§:** For this search a Mascot score ≥38 is significant (p<0.05).

e Sequence coverage is based on peptides with unique sequence.

f NCBI UniGene EST profile, TPM = Transcripts per million.

**Table 3 ppat-1000085-t003:** Cellular proteins in purified influenza virions identified only by MudPIT LC-MS/MS analysis.

		MudPIT Analysis				[Table-fn nt119]		
Protein Name	Entrez Gene ID	No. of observed peptides^a^	Protein prophet score^b^	sequence coverage (%)^c^	Cellular localization	Expression profile (TPM)^d^		Reported in other viruses
						Kidney	Lung	
**CD59**	966	3	1	27.8	membrane	293	271	HCMV [81], HTLV-1 [81], HIV-1 [80], VV [82]
**2′,3′-cyclic nucleotide 3′ phosphodiesterase**	1267	1	1	11.7	cytoplasm/membrane?	369	65	HIV-1 [11]
**ubiquitin carboxyl-terminal hydrolase L1**	7345	1	1	14.8	cytoplasm/membrane?	175	494	
**fatty acid synthase**	2194	1	1	5.7	cytoplasm	33	238	
**gamma-glutamyltransferase 1**	2678	2	1	4	membrane	4	23	HIV-1 [11]
**HSP 27 kDa**	3315	2	1	13.6	cytoplasm/nucleus	364	503	HIV-1 [90]
**WD repeat-containing protein 1**	9948	2	1	5.2	cytoplasm	639	288	
**phosphoglycerate kinase**	5230	1	0.99	15.9	cytoplasm	833	479	HIV-1 [11], HCMV [3]
**diazepam binding inhibitor**	1622	1	0.99	23.8	cytoplasm/membrane?	113	80	
**transgelin**	6876	1	0.98	6	cytoplasm	113	342	
**S100 calcium-binding protein A11**	6282	1	0.97	10.9	cytoplasm/membrane	174	243	HIV-1 [11]
**integrin beta 1**	3688	1	0.97	12.5	membrane	198	186	HIV-1[11], MoMLV[13]
**annexin A11**	311	1	0.9	2.2	cytoplasm/membrane	175	232	HIV-1 [11]

a Observed peptides include all peptides that differ either by sequence, modification or charge.

b A Protein prophet score ≥0.95 is significant (p<0.05).

c Sequence coverage is based on peptides with unique sequence.

d NCBI UniGene EST profile, TPM = Transcripts per million.

**Table 4:** Cellular proteins identified in purified influenza virions only by gel fractionation LC-MS/MS analysis.

			SDS-PAGE and LC-MS/MS Analysis					[Table-fn nt127]		
Protein Name	Entrez Gene ID	Mass (Da)	Gel slice^a^	No. of observed peptides^b^	MASCOT score^c^	Sequence coverage (%)^d^	Cellular localization	Expression profile (TPM)^e^		Reported in other viruses
								kidney	lung	
**aldo-keto reductase**	231	35854	29	5	101	23.1	cytoplasm	653	214	
**annexin A5**	308	35937	29*	5	226	15.6	cytoplasm/membrane	113	247	HCMV [3], HIV-1 [11]
**tropomyosin 4**	7171	28522	31	7	138	21.4	cytoplasm	108	83	
**peroxiredoxin 1**	5052	22110	39	4	85	19.6	cytoplasm	326	545	VV [10], HIV-1 [11], MoMLV [13]
**destrin**	11034	15397	45	4	113	32.6	cytoplasm	137	157	
**ubiquitin**	7314	8565	18#	2	39§	32.9	cytoplasm/nucleus	169	183	HIV-1 [11],[12],[91], SIV [91], MoMLV [13],[91], VV [10]

*: Best sequence coverage was obtained with the deglycosylated sample.

a Gel slices were numbered consecutively from the top to the bottom of an 8–16% gel.

# From a higher percentage gel.

b Observed peptides include all peptides that differ either by sequence, modification or charge.

c A Mascot score ≥50 is significant (p<0.05).

d Sequence coverage is based on peptides with unique sequence.

**§:** For this search a Mascot score ≥38 is significant (p<0.05).

e NCBI UniGene EST profile, TPM = Transcripts per million.

As with the viral proteins, comparison between the two MS methods reveals greater sequence coverage obtained with the gel-separated proteins, however in total more proteins were identified with the MudPIT analysis. Both cytoplasmic and membrane-bound proteins were identified and while several of these proteins are highly abundant according to their NCBI UniGene EST profiles, most do not fall into this category and are present at moderate or low abundance in the cell. It is also striking that the majority of the proteins, particularly those in Table 2 have been reported in other virus particles and that many proteins are related or can be grouped together in functional categories such as cytoskeletal components, glycolytic enzymes and annexins.

### Confirmation of cellular protein incorporation into influenza virions

**Figure 2:**
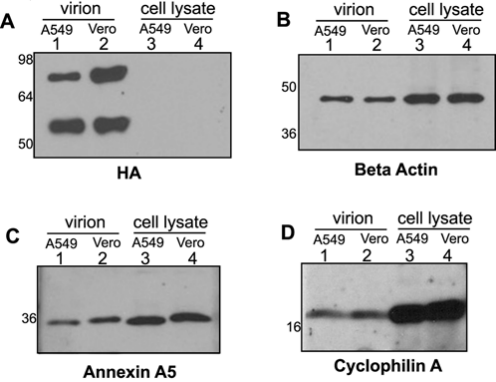
Confirmation of host protein incorporation into influenza virions derived from different cell lines. Influenza A/WSN/33 virus was purified from the supernatant of infected A549 and Vero cells. 2 ug of purified virus derived from A549 and Vero cells (lane 1 and 2, respectively) and 10 ug of cellular extracts from uninfected A549 and Vero cells (lanes 3 and 4, respectively) were subjected to western blot analysis with antibodies against the following proteins: (A) Influenza hemagglutinin (HA0 and HA1 are visible), (B) Beta actin, (C) Annexin A5, (D) Cyclophilin A. Numbers to the left are molecular weight markers.

Following identification of the cellular proteins by proteomic methods, their presence in the purified influenza virus preparation was verified by immunoblot analysis which provides the highest degree of specificity. Influenza virus preparations purified from both Vero and A549 cells were analyzed for the presence of HA, beta-actin, annexin A5 and cyclophilin A (Fig. 2). Extracts from uninfected Vero and A549 cells were included as a control for the reactivity of the antibodies and size of the cellular protein. Influenza virus purified from both cell lines showed the presence of these three cellular proteins, confirming that they are associated with the virus and that this can be demonstrated in virus grown in two different cell types.

**Figure 3:**
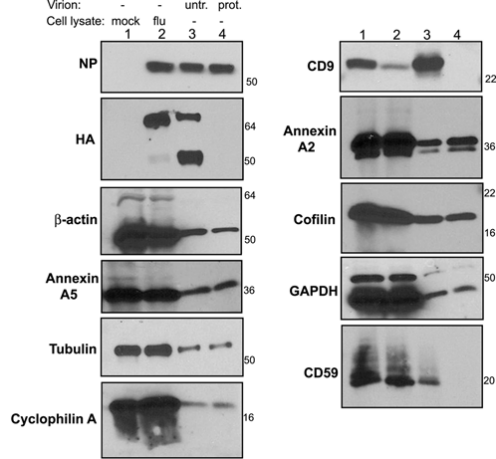
The effect of protease treatment on influenza virion associated host proteins. Purified influenza A/WSN/33 virus was either mock treated or subjected to overnight digestion with subtilisin followed by concentration through a sucrose cushion. 10 ug of mock infected cell lysate (lane 1) or influenza infected cell lysate (lane 2) and 2 ug of untreated influenza virions (lane 3) or protease treated influenza virions (lane 4) were then analyzed by western blot with antibodies against the indicated proteins. Numbers to the right are molecular weight markers.

**Figure 4:**
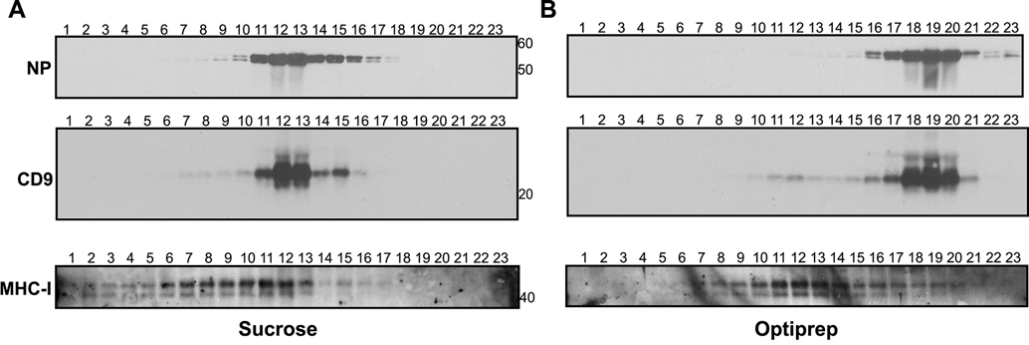
Gradient fractionation demonstrates co-purification of influenza virus and CD9. Influenza A/WSN/33 virus was purified over (A) sucrose and (B) Optiprep gradients. Fractions were taken from the top and analyzed by western blot for the presence of NP, CD9 and MHC-I, as indicated. Numbers to the right are molecular weight markers.

When analyzing the results of virion proteomic studies, the challenge is to prove that the cellular proteins are really an integral part of the virion and that they are not just attached non-specifically to the outside or are perhaps derived from a microvesicle or exosome that co-purified with the virus. To address this question, we used the subtilisin protease protection assay which has been shown to efficiently remove microvesicles from HIV-1 virion preparations [30],[31]. Protease treatment of the purified virus preparation strips proteins off the outside of virus particles and off any contaminating microvesicles. In doing so, the microvesicles become lighter than the virions and therefore the virions can be isolated by density centrifugation. Proteins that are inside the virion are protected by the lipid envelope and therefore will remain after the protease treatment. This is illustrated by the presence and absence of NP and HA, respectively, after subtilisin treatment of influenza virions (Fig. 3). Immunoblot analysis of selected cellular proteins reveals that beta-actin, annexin A5, tubulin, annexin A2, cofilin, GAPDH and cyclophilin A are all still present following protease treatment and centrifugation (Fig. 3). This indicates that these proteins are inside the influenza virion, however it should be noted that these experiments do not absolutely exclude the possibility that some proteins may be derived from contaminants that were not efficiently removed by the protease treatment. In contrast, CD9 and CD59 are absent following treatment (Fig. 3). There are two possible interpretations of this finding: Firstly, their loss may be because they are associated with microvesicles rather than virions and secondly, these proteins may be exposed on the surface of the virion as is HA. Since CD9 and CD59 are both membrane-bound proteins found on cellular surfaces (CD9 has two extracellular loops and CD59 is GPI-anchored), if they are incorporated into an influenza virus particle one would expect them to be in the viral envelope and thus sensitive to protease digestion. However, to further address the possibility that they are not part of the virion, we made use of an alternative gradient medium (Optiprep) which, unlike sucrose, maintains iso-osmotic conditions at high densities and is therefore particularly good at separating membranous organelles such as enveloped viruses and microvesicles. Influenza virus preparations were purified simultaneously over both sucrose and Optiprep gradients, which were then fractionated. Immunoblot analysis demonstrated that CD9 co-sediments precisely with influenza virus (as detected by the presence of NP) in both types of gradient (Fig. 4). We also examined the separation of MHC-I, which has been found in exosomes derived from a variety of cell types [32]–[34] but was not identified in the mass spectrometry analysis of purified influenza virus. In the sucrose gradient, the peak MHC-I staining overlaps partially with that of NP and CD9 but in the Optiprep gradient there is clear separation of MHC-I from virus and CD9. This supports the idea that Optiprep allows for better separation and strongly suggests that CD9 is an integral part of the influenza virion. It should also be noted that despite partial co-purification of MHC-I in the sucrose gradient, this protein was not identified in the proteomic analysis, probably indicating that the level of sensitivity provided by these methods is not sufficient to detect very low levels of protein.

**Figure 5:**
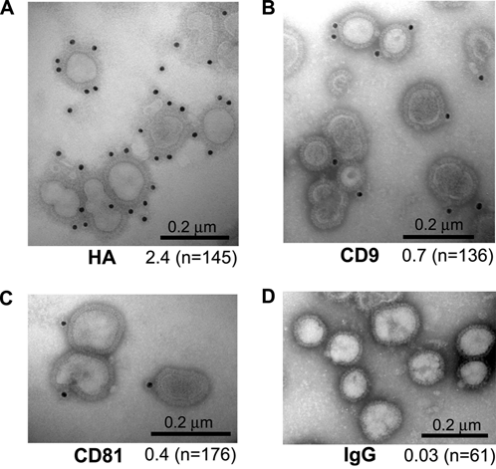
Immunogold labeling of host proteins in purified influenza virions. Influenza virions purified from the supernatant of infected Vero cells were immunogold labeled with antibodies against (A) Hemagglutinin, (B) CD9, (C) CD81 and (D) normal mouse IgG. Labeled virus was negatively stained with sodium silicotungstate and visualized by electron microscopy (50,000× magnification). The number of gold particles per virion is shown below (n = the number of virions counted).

To provide additional evidence that the membrane-bound cellular proteins identified by mass spectrometry are on the lipid envelope of influenza virus, immunogold labeling of Optiprep-purified influenza virions was performed. Virions were labeled with antibodies against HA, CD9, CD81 (Fig. 5) or CD59 (data not shown) and secondary gold antibodies, followed by negative staining. One or two gold particles located on the surface of a virion could be seen for CD9, CD81 and CD59. This was significantly less compared with the degree of HA labeling, however it is consistent with the fact that there is most likely far more HA present on the virions than there are molecules of CD9, CD81 or CD59.

## Discussion

Our proteomic analysis of influenza A virions has confirmed the presence of nine virus-encoded proteins in the virus particle and for the first time demonstrated the incorporation of cellular proteins. In total 36 host proteins were identified with a confidence level >95% based on matches of the peptide sequences with proteins in the NCBI database, and 17 of these were detected using two independent techniques. It is remarkable that of these 36 proteins, 25 have also been described to be present in virions of quite diverse virus families (e.g. herpesviruses, poxviruses, retroviruses–see Tables 2–4). Considering that these studies were performed independently using different cell types and different mass spectrometry methods, this similarity is probably not an issue of contamination. The most likely explanation is that these viruses all share some fundamental feature and that these host proteins are involved in the processes associated with that common trait. For instance it could simply be that they are all enveloped viruses. Enveloped viruses must all enter the cell via a membrane fusion event and exit by budding, be it from the plasma membrane or an internal membrane. Therefore one hypothesis would be that the incorporated host proteins common to all these enveloped viruses play a role in these particular stages of the virus life-cycle. Future experiments involving RNAi knockdown of these host proteins and also information on host factors associated with non-enveloped viruses will help to address this question. The process of budding does, of course, lend itself to the entrapment of proteins that are fortuitously at the budding site as the particle forms. Most likely, these would be highly abundant cytosolic proteins, and several of the proteins found within both influenza virus and other virus particles would fall into this category (e.g. beta actin, enolase, tubulin, GAPDH, pyruvate kinase). These proteins may be examples of non-specifically incorporated proteins. Other, less abundant, proteins may be incorporated because they are enriched at the virus budding site. Some viruses, including influenza virus and HIV-1 have been proposed to assemble at and bud from specific microdomains in the plasma membrane termed “lipid rafts” [35]–[39]. Lipid rafts are characterized as being rich in sphingolipids, cholesterol and specific raft proteins, and lipid analysis of purified HIV-1 virions has shown a composition strikingly similar to that of lipid rafts [40]. Therefore it would not be unexpected to find lipid raft-resident proteins within virus particles that bud from these domains. Proteomic analysis of rafts derived from a number of different cell types, including Vero cells, has been performed and comparison of these proteins with those that were found to be associated with influenza virions reveals some overlap [41]–[45]. These include tubulin, actin, annexins, enolase, GAPDH, glypican 4, gamma-glutamyltransferase, HSP 27 and transgelin. As a GPI-anchored protein, CD59 is also considered to be a lipid raft protein. It should be noted that some typical raft proteins such as caveolin and flotillin were not identified in influenza virus particles and the same is true for HIV-1 [11],[40]. It is thought that this is because the viral budding site is formed by the clustering of only a subset of rafts which may be determined by the accumulation of specific viral proteins [40]. The question remains as to whether the incorporation of these raft proteins is secondary to the choice of budding site or whether the budding site is selected due to the localized concentration of these proteins.

Some cellular proteins may be specifically recruited and packaged into the virion, presumably via an interaction with either a viral protein or even the viral genome. There is a high probability that such proteins are actively involved in the virus life cycle, either at late stages during virus assembly and egress from the producer cell or at early stages of entry into the new target cell. A number of the proteins identified in influenza virions have been reported to play a role during certain stages of the infection process for a variety of viruses and this may provide a clue as to why they are present in the influenza virion. These include:

### i) Cytoskeletal proteins

The host cytoskeletal network is involved in the transport of viral components in the cell and particularly during the stages of virus entry and exit [46],[47]. Several studies on RNA viruses have also indicated that cytoskeletal proteins such as tubulin and actin are required for viral gene expression [48]–[51]. For influenza virus, it has been shown that the virus requires an intact actin cytoskeleton for entry, specifically into polarized cells [52] and interactions between the cytoskeleton and lipid rafts has been proposed to facilitate budding of filamentous virus particles [53]. Furthermore, an association of M1 and NP with cytoskeletal elements has been reported [54],[55], and actin and tubulin were both identified as proteins that interact with influenza RNPs [21]. In the present study, protease treatment showed that actin, tubulin and cofilin (which binds to actin) were all present in the interior of influenza virions which most likely reflects their active participation in moving the viral components to the assembly site as well as cytoskeletal reorganization that occurs during bud formation. Other actin-binding proteins found to be associated with influenza virions are tropomyosin, annexin (see below), WD repeat containing protein and destrin.

### ii) Annexins

Several annexin family members (A1, A2, A4, A5 and A11) were identified in influenza virus particles. Annexins are calcium-dependent phospholipid-binding proteins and are proposed to act as scaffolding proteins at certain membrane domains. Annexin A2 in particular has been shown to bind to actin and be involved in the assembly of actin at cellular membranes [56]. It is also required for the apical transport of vesicles in polarized cells and specifically vesicles that carry membrane raft-associated proteins [57]. This is intriguing since influenza virus also buds from raft domains at the apical surface of polarized cells. In fact, a role for annexin A2 in virus assembly has been proposed for HIV-1 [58], and in HCMV, the presence of annexin A2 is thought to promote viral binding and fusion [59]. Interestingly, annexins A1 and A5, which both interact with A2, have the opposite effect of preventing fusion, perhaps indicating a potential regulatory role [60]. The calcium-binding protein S100A11 which is known to interact with annexin A1 [61] was also identified in the influenza virion, suggesting that they may be packaged as a complex.

### iii) Tetraspanins

Two members of the tetraspanin family, CD9 and CD81, were found to be associated with influenza virions and are most likely inserted into the viral envelope. Tetraspanins have four transmembrane domains and two extracellular loops and are involved in both homo- and heterotypic interactions in specialized membrane domains referred to as tetraspanin-enriched microdomains (TEMs) [62]. Despite some similarities to lipid rafts, proteomic analyses of TEMs and lipid rafts have shown that they have distinct compositions [63], although they may interact with each other under certain conditions. Several tetraspanins have been reported to play a role during viral infections. Of these, CD81 is the best characterized in terms of its function as a co-receptor for hepatitis C virus [64],[65]. Tetraspanins, including CD9 and CD81, have also been implicated in both fusion and egress pathways for a number of viruses such as HIV-1, feline immunodeficiency virus and canine distemper virus [66]–[70]. One such study also reported that in contrast to HIV, influenza virus does not assemble at domains rich in tetraspanins and does not incorporate either CD9 or CD63 into virus particles [71]. This finding is obviously contradictory to the present proteomic analysis of influenza virions in which CD9 was detected by mass spectrometry, immunoblot analysis and immunogold labeling of virions. The reason for the discrepancy may be technical as Khurana *et al.* [71] used HeLa cells to propagate the virus and detected incorporated proteins by immunofluorescent staining of concentrated virions. Integrin beta-1 was also identified in influenza virus particles and as integrins are well-characterized tetraspanin binding partners, it was possibly incorporated together with CD9 or CD81.

### iv) Cyclophilin A

Cyclophilin A (CypA), which was shown to be in the core of the influenza virion, is a peptidyl-prolyl isomerase and has been reported to be present in the virions of a number of different viruses. For HIV-1, the specific incorporation of CypA is mediated by an interaction with the capsid portion of the Gag protein [15],[16]. There is an abundant amount of literature concerning the requirement of CypA for HIV-1 infectivity but as it turns out, it is the CypA in the target cell that is more critical and therefore the precise role of the virion CypA is currently unclear [72],[73]. Within the target cell, CypA is proposed to facilitate a conformational change in the capsid which enables the virus to evade detection by the host immune response [74],[75]. CypA is incorporated into vesicular stomatitis virus (VSV) presumably via the described interaction with the nucleocapsid protein [76]. It has also been shown to be required for VSV replication, however this activity is serotype-specific [76]. A strong interaction between CypA and SARS coronavirus nucleocapsid protein has also been reported [77] and CypA relocalizes to sites of viral replication in vaccinia virus infected cells [78]. Another member of the cyclophilin family, cyclophilin B, is required for hepatitis C virus replication and acts by interacting with the viral polymerase and increasing its RNA binding activity [79]. Therefore, there is a strong precedent for the involvement of cyclophilin proteins in virus replication.

### v) CD59

CD59 is a complement regulatory protein that acts by inhibiting formation of the membrane attack complex (MAC). It is a GPI-anchored protein and the experimental data confirm that it is associated with the influenza virus envelope. Enveloped viruses are susceptible to direct complement-mediated lysis by MAC and as a form of protection HIV-1, vaccinia virus (VV), human T cell lymphotropic virus (HTLV) and human cytomegalovirus (HCMV) all incorporate CD59 and other regulatory proteins such as DAF and CD46 into their lipid envelopes (the latter two were not identified in influenza virions) [80]–[82]. Complement control proteins are highly species specific and are only active against homologous complement. This has important implications for virus host-range as the virus produced and transmitted between one host species would be protected by incorporation of CD59/DAF/CD46, however, virus transmitted to another host species would become susceptible to lysis by the complement system of that host.

### vi) Glycolytic enzymes

When one looks at the list of proteins associated with influenza virions, at first glance it is difficult to see an obvious role for some of these proteins in the virus life cycle. However, it is possible that some of these cellular proteins have functions other than their described major roles. For example, a number of proteins involved in the glycolytic pathway were identified (pyruvate kinase, enolase 1, GAPDH, phosphoglycerate kinase). Both enolase and phosphoglycerate kinase, in addition to tubulin, have been reported to stimulate transcription of the Sendai virus genome [83], but it is unclear whether their glycolytic activities are required or whether this is an example of an alternative function for these proteins [84]. A role in RNA virus transcription has also been proposed for GAPDH. Phosphorylated forms of GAPDH have been shown to bind to the genomic *cis*-acting RNA of human parainfluenza virus type 3 (hPIV3) and are also present in purified virions [85],[86]. *In vitro* data indicate that GAPDH serves a negative regulatory role in hPIV3 transcription and that this is dependent on its phosphorylation [85].

Compared with the cellular proteins found to associate with the influenza RNP complex, the only ones also identified in influenza virions are alpha and beta tubulins, beta actin and ubiquitin carboxyl-terminal hydrolase [21]. This may indicate that these proteins are packaged with RNPs and that they interact with one of the RNP components i.e. NP, one of the polymerase proteins or the genomic RNA. The fact that there are not more proteins in common is probably because each viral protein associates with many different cellular proteins during the course of the viral life cycle and these interactions in most cases are transient. The proteins identified in this and other studies represent a snapshot of a particular point in the life cycle, but importantly they provide a foundation for further analysis of cellular requirements for influenza virus infection. Packaged cellular proteins have a unique importance as the virus literally transports them from one cell to the next. This is an ingenious way of ensuring that host cell activities required at or immediately after entry are instantly accessible to the virus. For viruses that can infect multiple species such as influenza virus, any host protein that is required for infection must be active in both species to allow for transmission to occur. Therefore, as discussed above for CD59, virion-associated host proteins can be one of the determinants of virus host range due to their species-specific activity. It will also be interesting to compare the identity and abundance of host proteins in influenza viruses that produce virions with a filamentous morphology. One would assume that the increased volume and surface area of these particles would allow for greater levels of host protein incorporation but whether or not there is increased diversity may depend on specific versus non-specific incorporation.

The presence of host proteins in influenza virions, whether they are incorporated specifically or non-specifically, could also be a concern for vaccine manufacturers as the vaccine is delivering more than just viral antigens. Although the relative amount of cellular protein compared to viral protein in the virus particle is expected to be extremely small, the choice of host cell for propagation of vaccines could be an important consideration, particularly for live-attenuated virus vaccines. Currently, all influenza vaccines are produced in embryonated chicken eggs but there is a move afoot to transition to cell culture systems, with Vero cells being one of the approved cell lines [87],[88]. During the manufacturing process great care is taken to avoid the use of animal-derived products such as serum but the incorporation of non-human primate proteins into the vaccine virus will be unavoidable. Precise quantitation of these non-viral components will help to assess whether the levels present in each vaccine dose are high enough to risk inducing an allergic response.

## Materials and Methods

### Cells, virus and antibodies

Vero and A549 cells were maintained in Dulbecco's modified Eagle medium (Gibco, San Diego, California) supplemented with 10% fetal bovine serum (HyClone, South Logan, Utah) and Madin-Darby canine kidney (MDCK) cells were maintained in minimal essential medium supplemented with 10% fetal bovine serum. Influenza A/WSN/33 virus was propagated in MDCK cells in Minimal Essential Medium (Gibco) supplemented with 0.3% bovine serum albumin (Sigma, St. Louis, Missouri) and 0.1% fetal bovine serum. Viral titers were determined by plaque assay on MDCK cells.

Antibodies against actin (A4700), annexin A5 (A8604), cofilin (C8736) and tubulin (T0198) were obtained from Sigma (St. Louis, Missouri). Monoclonal antibody against annexin A2 (sc-28385) was obtained from Santa Cruz Biotechnology (Santa Cruz, California). Monoclonal antibodies against CD9 (sc-13118 and 555370) were obtained from Santa Cruz Biotechnology and BD Pharmingen (San Diego, California), respectively. Monoclonal antibody against CD59 (MCA1054GA) was obtained from Serotec (Oxford, U.K.) and monoclonal antibody against CD81 (555675) was obtained from BD Pharmingen. Rabbit polyclonal antibody against cyclophilin A (SA-296) was obtained from Biomol (Plymouth Meeting, Pennsylvania) and monoclonal antibody against GAPDH (RDI-TRK5G4-6C5) was obtained from RDI Research Diagnostics (Concord, Massachusetts). Monoclonal antibodies against influenza virus NP (HT103) and HA (2G9) were made by the Mount Sinai Hybridoma Center Shared Research Facility. The MHC-I antibody was kindly provided by Dr. Domenico Tortorella (Mount Sinai School of Medicine, NY).

### Purification of influenza virus

Fifty 15cm dishes of 80% confluent Vero cells were infected with influenza A/WSN/33 virus at a multiplicity of 0.001. At 65–70 hours post infection, the supernatant was harvested and clarified (2600×*g*, 5 min, 4°C, in a Sorvall RT6000D centrifuge). The clarified supernatant was layered over a 20% sucrose cushion in NTE buffer (100 mM NaCl, 10 mM Tris-Cl (pH 7.4), 1 mM EDTA) and the virus concentrated by ultracentrifugation (112,600×*g*, 2 hrs, 4°C, in a SW28 rotor [Beckman Coulter, Fullerton, California]). The concentrated virus was purified over a 30–60% sucrose gradient (112,600×*g*, 3 hrs, 4°C) and the banded virus collected, diluted with NTE buffer, pelleted (112,600×*g*, 90 min, 4°C) and resuspended in approximately 1 ml of NTE buffer. Typical protein yields of 1–2 mg/ml were obtained. When using Optiprep medium (Sigma, St. Louis, Missouri), a 10–30% gradient was made and fractions were taken from the top. Protein was precipitated from each fraction with 20% trichloroacetic acid (TCA) and subjected to western blot analysis.

### Deglycosylation of virion proteins

Purified virus equivalent to 100 ug of protein was denatured by heating at 100°C for 10 min in the presence of 0.5% SDS, 40 mM DTT and 1%NP40. PNGase F (New England Biolabs, Ipswich, Massachusetts) was added in the presence of 50 mM sodium phosphate (pH 7.5) and 1% NP40 and the reaction incubated at 37°C overnight.

### Protease treatment of virions

Purified virus equivalent to 50 ug of protein was incubated with 100ug of subtilisin protease (Sigma, St. Louis, Missouri) in 20 mM Tris-Cl (pH 8) and 1 mM CaCl_2_ for 18 hours at 37°C. The treated virus was diluted to 1 ml with NTE buffer and 5ug of PMSF (Sigma) was added. The virus was concentrated through a 20% sucrose cushion by ultracentrifugation (222,030×*g*, 2 hr, 4°C in an SW41 rotor [Beckman Coulter, Fullerton, California]) and then subjected to western blot analysis.

### Protein gel electrophoresis and Immunoblot analysis

Vero or A549 cells at 80% confluency were mock infected or infected with influenza A/WSN/33 virus at a multiplicity of 0.001. At 65–70 hours post infection the cells were harvested and whole cell extracts were prepared by lysis in extract buffer (50 mM Tris [pH 7.5], 280 mM NaCl, 1% Triton X-100, 0.2 mM EDTA, 2 mM EGTA, 10% glycerol, 1 mM dithiothreitol, 0.1 mM sodium vanadate and protease inhibitors [Complete; Roche]) on ice for 30 minutes. Extracts were centrifuged (15700×*g*, 15 min, 4°C in an Eppendorf 5415R microcentrifuge) and the supernatants collected. Proteins from either purified virus (2 ug) or whole cell extracts (10 ug) were denatured by heating at 100°C for 10 min in 1× sodium dodecyl sulfate-polyacrylamide gel electrophoresis (SDS-PAGE) sample buffer and were then separated by SDS-PAGE. For western blot analysis the proteins were transferred to nitrocellulose membrane which was then probed with a specific primary antibody and a peroxidase-labeled secondary antibody. The blots were analyzed by chemiluminescence and exposed to x-ray film. For protein staining, gels were stained with SimplyBlue SafeStain (Invitrogen, Carlsbad, California).

### Electron microscopy and Immunogold labeling

Optiprep-purified virus was diluted 1∶20 with NTE buffer and adsorbed onto formvar/carbon-coated nickel grids (Electron Microscopy Sciences, Hatfield, Pennsylvania). Following a 5 min wash with TBS buffer (50 mM Tris-Cl (pH 7.5), 150 mM NaCl), the sample was blocked with 3%BSA in TBS for 45 min. Primary antibody (10 ug/ml) was diluted in 1%BSA/TBS and adsorbed to the grid for 1 hr at room temperature. Following three washes with TBS, secondary gold-conjugated antibody was added for 1 hr at room temperature. The grids were then washed twice with TBS, once with water and negatively stained with 1% sodium silicotungstate (pH 7) for 15 sec. Images of stained virions were captured on a Hitachi H-7650 120 kV transmission electron microscope. For quantitation purposes, the number of virions and the number of gold particles were assessed in two representative images. These data were expressed as the number of gold particles per virion.

### Protein identification from gel slices

Proteins separated in one dimensional polyacrylamide gels were cut sequentially and subjected to *in situ* tryptic digestion prior to mass spectrometric analysis. Digestion was performed robotically on the GE Healthcare Ettan Gel Digester in a 96 well plate. A 20 minute wash with 100 µl, 50 mM ammonium bicarbonate in 50% acetonitrile was followed with a 10 minute 75% acetonitrile wash. Gel bands were then air dried and 15 µl of 6.7 µg/µl sequencing grade trypsin (Promega) was added to each well. Digestion was carried out at 37°C for 16 hours. The protein digests were then analyzed using Waters/Micromass QTOF Ultima mass spectrometer equipped with a Waters CapLC liquid chromatography system. 10 µl of the digest supernatant was loaded into a capLC vial and 5 µl of the sample was directly injected onto a 100 µm i.d.×150 mm long Atlantis C18 reversed phase column (Waters) running at 500 nl/min. Initial HPLC conditions were 95% buffer A and 5% buffer B with the following linear gradient: 3 min, 5% B; 43 min 37% B; 75 min 75% B; and 85 min 95% B. Buffer A consisted of 98% water, 2% acetonitrile, 0.1% acetic acid, and 0.01% TFA. Buffer B contained 80% acetonitrile, 20% water, 0.09% acetic acid, and 0.01% TFA. Data-dependent acquisition was performed so that the mass spectrometer switched automatically from MS to MS/MS modes when the total ion current increased above the 1.5 counts/second threshold set point. In order to obtain good fragmentation, a collision energy ramp was set for the different mass sizes and charge states, giving preference to double- and triple-charged species for fragmentation.

All raw MS/MS spectral data were searched in-house using the MASCOT algorithm (Matrixscience) with the Mascot Distiller program utilized to generate Mascot compatible files. The Mascot Distiller program combines and centroids sequential MS/MS scans from profile data that have the same precursor ion. A charge state of +2 and +3 was preferentially located with a signal to noise ratio of 1.2 or greater and a peak list was generated for database searching. Using the Mascot database search algorithm, a protein was considered identified when Mascot listed it as a significant match/score (p<0.05) with the proper enzymatic cleavage sites. Unlike the MudPIT analysis (see below), the Peptide/Protein Prophet (Institute for Systems Biology) scoring system was not used here because this would have required either combining the data from all gel slices or treating each gel slice as an individual Peptide/Protein Prophet model. Combining the gel slices may allow for an effective PeptideProphet expectation maximization model to be built but would create false protein identifications in that a protein probability could be based on peptides present in separate bands on the gel. Applying Peptide/Protein Prophet to individual gel slices would result in a collection of small datasets (50–100 MS/MS queries) that cannot be modeled accurately as there are not sufficient datapoints for the expectation maximization algorithm to assign correct versus incorrect peptides.

The NCBInr protein database was chosen over other genome specific databases to allow a wider search match found based on homology to other species. Parameters used for searching were partial methionine oxidation and acrylamide modified cysteine, a peptide tolerance of ±0.6 Da, and a MS/MS fragment tolerance of ±0.4 Da.

### MudPIT protein identification

100 µg of deglycosylated purified virus preparation was solubilized in 8 M Urea, 0.4 M NH_4_HCO_3_ (pH 8.0), reduced with 45 mM DTT, and alkylated with 100 mM iodoacetamide. Tryptic digestion was performed using a 1∶50 enzyme to substrate ratio at 37 degrees C for 18–24 hours (sequencing grade trypsin, Promega). After digestion, off-line strong cation exchange chromatography (SCX) was performed on an Applied Biosystems Vision Workstation using a 2.1 mm×200 mm PolySulfoethyl A column, equilibrated with Buffer A (10 mM KH_2_PO_4_, 25% Acetonitrile, pH = 3.0). Peptides were separated into fractions using a 90 min linear salt gradient from 0–98% Buffer B (10 mM KH_2_PO_4_, 25% Acetonitrile, 1 M KCl, pH = 3.0) All 22 collected fractions from the SCX chromatography were dried and reconstituted with 15 µl of 0.1 % TFA. A 5 µl aliquot of each of the samples was injected and desalted on a reversed phase C18 trap column (Waters, Symmetry, Nanoease 0.180 mm i.d.×23.5 mm, 5 micron) and was separated on a C18 analytical column (Waters, Atlantis, Nanoease 0.1 mm i.d.×150 mm, 3 micron, 100 Å) using the Dionex Ultimate chromatography system. On-line MS analysis was performed on the ABI QSTAR XL system. MS data was surveyed for 0.5 s, and MS/MS acquisition was performed on three highest peptide peaks.

Each of the QSTAR XL mass spectrometer spectra files was processed with MASCOT Distiller version 2.1 and the resulting peak lists were database searched using MASCOT Server 2.1. The search parameters included static carbamidomethyl modifications for cysteine and variable oxidation modifications for methionine amino acid residues. Data analysis on the resulting LC/MS and MS/MS datasets is accomplished using a dual processor Dell 650 Workstation. The search results for each fraction were analyzed using the NCBInr database. After MASCOT analysis, Peptide and ProteinProphet (Institute for Systems Biology) analysis was performed using the Trans-Proteomic Pipeline version 2.9 GALE rev.1, Build 200607201423. Peptide and Protein Prophet computes the probabilities for both individually searched peptides and the resulting proteins. The 95% Protein Prophet probability cutoff corresponds to a 0.6% false positive error rate. Finally, TPP identifications are submitted to Yale Proteomics Expression Database (YPED) web site [89] for further user analysis. All data are publicly available through http://yped.med.yale.edu/repository.

## Supporting Information

Table S1 Comparison of viral proteins identified by gel fractionation LC-MS/MS in glycosylated and deglycosylated influenza virions.
(0.05 MB DOC)

Table S2 Comparison of cellular proteins identified by gel fractionation LC-MS/MS in glycosylated and deglycosylated influenza virions.
(0.09 MB DOC)
