# Supplementary material for: Cellular Proteins in Influenza Virus Particles
Source: PLoS Pathog. 2008 Jun 6;4(6):e1000085. doi: 10.1371/journal.ppat.1000085 (PMC2390764; doi:10.1371/journal.ppat.1000085)
Supplement: Table S1 — Comparison of viral proteins identified by gel fractionation LC-MS/MS in glycosylated and deglycosylated influenza virions. (0.05 MB DOC) [file ppat.1000085.s001.doc]

TABLE S1. Comparison of viral proteins identified by gel fractionation LC-MS/MS in glycosylated and deglycosylated influenza virions.

|  | | **GLYCOSYLATED** | | | | **DEGLYCOSYLATED** | | | |
| --- | --- | --- | --- | --- | --- | --- | --- | --- | --- |
| Protein Name | Mass (Da) | Gel slice*^a^* | No. of observed peptides*^b^* | Mascot score*^d^* | Sequence coverage (%)*^e^* | Gel slice*^a^* | No. of observed peptides*^b^* | Mascot score*^d^* | Sequence coverage (%)*^e^* |
|  |  |  |  |  |  |  |  |  |  |
| **PB1** | 86516 | 10 | 35 | 700 | 37.1 | 9 | 28 | 580 | 27.2 |
|  |  |  |  |  |  |  |  |  |  |
| **PB2** | 85796 | 11 | 34 | 768 | 35.6 | 10 | 26 | 742 | 30.6 |
|  |  |  |  |  |  |  |  |  |  |
| **PA** | 82531 | 11 | 23 | 458 | 28.2 | 10 | 18 | 460 | 20.8 |
|  |  |  |  |  |  |  |  |  |  |
| **HA** | 63525 | 11-17,21-24,26-34,38-43,48 | 2-70*^c^* | 54-546*^c^* | 29.4 | 3,4,9,10,12-21,23-41,43-47 | 2-121*^c^* | 53-494*^c^* | 19.1 |
|  |  |  |  |  |  |  |  |  |  |
| **NP** | 56244 | 15-19,23,27,30 | 5-61*^c^* | 66-1073*^c^* | 46.8 | 3,6-25,29 | 2-51*^c^* | 76-1044*^c^* | 48.8 |
|  |  |  |  |  |  |  |  |  |  |
| **NA** | 49689 | 20-21,28 | 3-6*^c^* | 52-98*^c^* | 15.5 | 19-21 | 2-7*^c^* | 94-159*^c^* | 9.1 |
|  |  |  |  |  |  |  |  |  |  |
| **M1** | 27864 | 32-46 | 3-102*^c^* | 76-787*^c^* | 66.3 | 12,18,19,28,29,31-37,39-44 | 3-63*^c^* | 61-697*^c^* | 65.5 |
|  |  |  |  |  |  |  |  |  |  |
| **M2** | 11313 | 46-47 | 2-3*^c^* | 39-101*^c^* | 48.5 | ND |  |  |  |

ND – not detected

*^a^* Gel slices were numbered consecutively from top to bottom of the 8-16% gel shown in Fig. 1B.

*^b^* Observed peptides include all peptides that differ either by sequence, modification or charge.

*^c^* Values represent the range when the protein was found in multiple gel slices.

*^d^* A Mascot score ≥ 50 is significant (p < 0.05).

*^e^* Sequence coverage is based on peptides with unique sequence.
