# Supplementary material for: Cellular Proteins in Influenza Virus Particles
Source: PLoS Pathog. 2008 Jun 6;4(6):e1000085. doi: 10.1371/journal.ppat.1000085 (PMC2390764; doi:10.1371/journal.ppat.1000085)
Supplement: Table S2 — Comparison of cellular proteins identified by gel fractionation LC-MS/MS in glycosylated and deglycosylated influenza virions. (0.09 MB DOC) [file ppat.1000085.s002.doc]

TABLE S2. Comparison of cellular proteins identified by gel fractionation LC-MS/MS in glycosylated and deglycosylated influenza virions.

|  | | **GLYCOSYLATED** | | | | **DEGLYCOSYLATED** | | | |
| --- | --- | --- | --- | --- | --- | --- | --- | --- | --- |
| Protein Name | Mass (Da) | Gel slice*^a^* | No. of observed peptides*^b^* | Mascot score*^d^* | Sequence coverage (%)*^e^* | Gel slice*^a^* | No. of observed peptides*^b^* | Mascot score*^d^* | Sequence coverage (%)*^e^* |
|  |  |  |  |  |  |  |  |  |  |
| **pyruvate kinase** | 57878 | 17 | 6 | 78 | 11.9 | ND |  |  |  |
|  |  |  |  |  |  |  |  |  |  |
| **alpha tubulin** | 50158 | 20 | 1 | 58 | 2 | 18-19*^c^* | 3-7*^c^* | 186-314*^c^* | 20.6 |
|  |  |  |  |  |  |  |  |  |  |
| **beta tubulin** | 47767 | ND |  |  |  | 19 | 27 | 555 | 35.9 |
|  |  |  |  |  |  |  |  |  |  |
| **enolase 1** | 47169 | 21 | 4 | 99 | 9.9 | 20 | 6 | 318 | 22.1 |
|  |  |  |  |  |  |  |  |  |  |
| **beta actin** | 41005 | 23 | 17 | 271 | 31.5 | 22-23 | 5-14*^c^* | 158-372*^c^* | 37.5 |
|  |  |  |  |  |  |  |  |  |  |
| **annexin A1** | 38714 | 28 | 6 | 90 | 21.7 | ND |  |  |  |
|  |  |  |  |  |  |  |  |  |  |
| **glyceraldehyde-3-phosphate dehydrogenase** | 36054 | 28 | 5 | 69 | 17.9 | ND |  |  |  |
|  |  |  |  |  |  |  |  |  |  |
| **annexin A2** | 38576 | 29 | 17 | 600 | 46.3 | ND |  |  |  |
|  |  |  |  |  |  |  |  |  |  |
| **annexin A5** | 35937 | ND |  |  |  | 29 | 5 | 226 | 15.6 |
|  |  |  |  |  |  |  |  |  |  |
| **tropomyosin 1** | 32876 | 29-30 | 3-10*^c^* | 75-233*^c^* | 34.2 | ND |  |  |  |
|  |  |  |  |  |  |  |  |  |  |
| **aldo-keto reductase** | 35854 | 29 | 5 | 101 | 23.1 | ND |  |  |  |
|  |  |  |  |  |  |  |  |  |  |
| **glypican 4** | 62398 | 30 | 3 | 63 | 5 | ND |  |  |  |
|  |  |  |  |  |  |  |  |  |  |
| **tropomyosin 3** | 27175 | 31 | 14 | 357 | 37.5 | ND |  |  |  |
|  |  |  |  |  |  |  |  |  |  |
| **tropomyosin 4** | 28522 | 31 | 7 | 138 | 21.4 | ND |  |  |  |
|  |  |  |  |  |  |  |  |  |  |
| **annexin A4** | 35883 | 31 | 10 | 181 | 39.5 | 30 | 2 | 196 | 10 |
|  |  |  |  |  |  |  |  |  |  |
| **CD9 antigen** | 25431 | 39 | 4 | 94 | 4.4 | 39 | 5 | 74 | 7.5 |
|  |  |  |  |  |  |  |  |  |  |
| **peroxiredoxin 1** | 22110 | 39 | 4 | 85 | 19.6 | ND |  |  |  |
|  |  |  |  |  |  |  |  |  |  |
| **cofilin 1** | 18502 | 44 | 11 | 318 | 50.6 | 43 | 13 | 354 | 48.8 |
|  |  |  |  |  |  |  |  |  |  |
| **destrin** | 15397 | 45 | 4 | 113 | 32.6 | ND |  |  |  |

ND – not detected

*^a^* Gel slices were numbered consecutively from top to bottom of the 8-16% gel shown in Fig. 1B.

*^b^* Observed peptides include all peptides that differ either by sequence, modification or charge.

*^c^* Values represent the range when the protein was found in multiple gel slices.

*^d^* A Mascot score ≥ 50 is significant (p < 0.05).

*^e^* Sequence coverage is based on peptides with unique sequence.
